# microbiONT: an AI-assisted, privacy-focused platform for local Nanopore 16S and 18S amplicon analysis

**DOI:** 10.1093/bioadv/vbag229

**Published:** 2026-08-12

**Authors:** Che-Chun Chen, Hsin-Yun Lu, Ying-Ning Ho

**Affiliations:** Institute of Marine Biology, National Taiwan Ocean University, Keelung, Taiwan; Department of Aquaculture, National Taiwan Ocean University, Keelung, Taiwan; Taiwan Oceans Genome Center, National Taiwan Ocean University, Keelung, Taiwan; Institute of Marine Biology, National Taiwan Ocean University, Keelung, Taiwan; Institute of Marine Biology, National Taiwan Ocean University, Keelung, Taiwan; Taiwan Oceans Genome Center, National Taiwan Ocean University, Keelung, Taiwan; Center of Excellence for the Oceans, National Taiwan Ocean University, Keelung, Taiwan

## Abstract

**Motivation:**

The affordability and high-throughput capabilities of Oxford Nanopore Technologies (ONT) have democratized genomic sequencing, yet the complexity of bioinformatics environment setup and analysis remains a barrier for many biologists.

**Results:**

microbiONT addresses this gap by providing a user-friendly, privacy-focused platform that features a streamlined, one-command installation process. Integrating a local Large Language Model (Llama 3.1) with an intuitive graphical interface, microbiONT serves not only as an analysis tool but also as an AI copilot to assist users in learning bioinformatics concepts. By translating natural language requests into executable commands and simplifying deployment, microbiONT empowers non-experts to perform rigorous or custom 16S/18S amplicon analysis locally. This ensures rapid data insights without the need for command line use. Beyond its AI-integrated GUI, the novelty of microbiONT lies in its fully localized, highly accurate, and privacy-preserving architecture for flexible data analysis.

**Availability and implementation:**

Source code and portable binaries are freely available at https://github.com/MBEBlab/microbiONT under the MIT license.

## 1 Introduction

Oxford Nanopore Technologies (ONT) has revolutionized microbial genomics by offering accessible third-generation sequencing. However, for researchers without specialized bioinformatics training, the steep learning curve of command-line tools and the complexities involved in selecting appropriate software and optimizing analysis parameters remain significant hurdles.

Commercial cloud-based solutions, while simplifying the analytical process, often necessitate high-bandwidth internet connectivity, incur additional operational costs, and present concerns regarding data privacy and security. Currently, only a limited number of bioinformatics workflows specifically tailored for Nanopore 16S amplicon analysis have been published, such as NanoCLUST and SituSeq (Santos *et al.* 2020, Rodríguez-Pérez *et al.* 2021, Zorz *et al.* 2023). In terms of user-friendly graphical interfaces, the primary option available is the official EPI2ME platform (https://epi2me.nanoporetech.com/). However, while EPI2ME streamlines the analytical process, it affords limited adaptability for optimizing specific parameters (e.g. minimum Q-score thresholds) or deploying custom, primer-specific reference databases. Furthermore, EPI2ME relies on a read-by-read classification strategy that is often insufficient to mitigate the inherent error rates of the underlying technology, leading to a high percentage of misclassified reads and increased uncertainty in taxonomic assignments.

To democratize access to these powerful tools, we present microbiONT. Unlike traditional pipelines that require intricate configuration, microbiONT prioritizes ease of deployment and usage. It is a local, privacy-focused platform that combines a user-friendly Streamlit interface with a local AI assistant. This hybrid approach allows users to perform basecalling, filtering, demultiplexing, and quality control via a standardized workflow. Uniquely, the integrated AI copilot transforms the analysis into an interactive learning opportunity by explaining parameters and logic. By removing technical barriers from installation to interpretation, microbiONT empowers wet-lab biologists to independently analyze genomic data on their local machines with confidence.

## 2 Methods

### 2.1 Architecture and design

**Figure 1:**
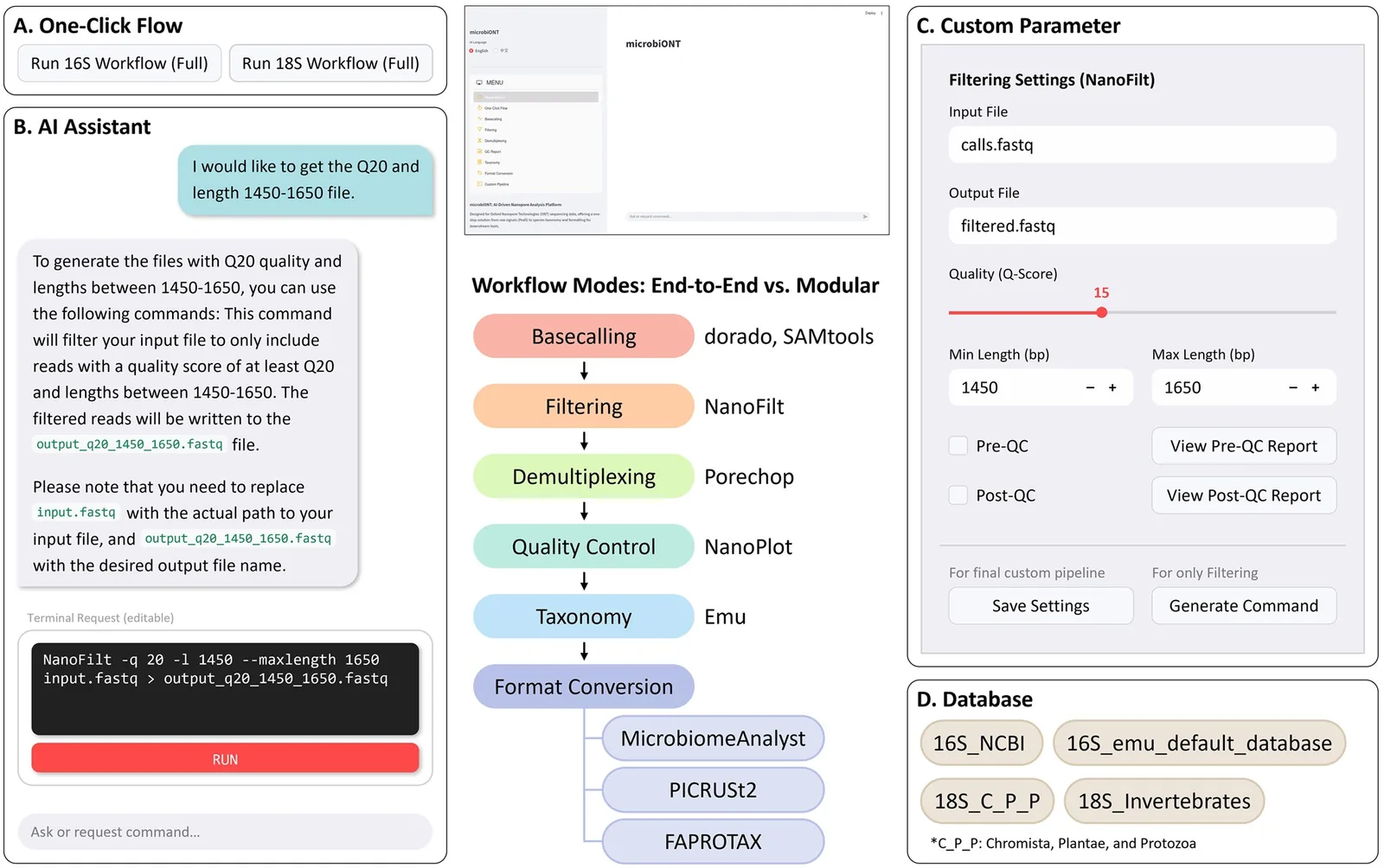
The general architecture of microbiONT. Overview of the integrated analysis pipeline and user interface components. (A) Automated “One-Click” workflows for routine analysis; (B) An interactive AI Assistant for natural language guidance and code generation; (C) Modular customization options for adjusting specific analysis parameters; and (D) A selection of 16S or 18S rRNA gene reference databases. The central flowchart illustrates the standardized processing steps from basecalling to format conversion utilizing established bioinformatics tools.

The microbiONT package is developed using Python and employs the Streamlit (https://github.com/streamlit/streamlit) framework for its graphical user interface (GUI). The platform is designed with a local-execution architecture, ensuring that all computational tasks, including sequencing data analysis and AI inference, are conducted exclusively on the host machine. This design prioritizes data privacy and operational capability in offline environments. The system utilizes Python’s subprocess module to manage the execution of underlying bioinformatics binaries, offering a unified interface that integrates command-line tools into a graphical environment. The general architecture of microbiONT is illustrated in Fig. 1.

### 2.2 Standardized analysis workflow

The primary analytical function is accessible through a standardized procedure that has been tailored for routine 16S/18S amplicon sequencing. This automated pipeline executes a predetermined sequence of processing steps (Fig. 1): (i) Basecalling: Raw Pod5 files are processed using Dorado v1.1.1 (https://github.com/nanoporetech/dorado), utilizing the SUP (Super-accurate) model to enhance sequence accuracy. The resulting BAM files are converted to FASTQ format using SAMtools v1.22.0 (Li *et al.* 2009). (ii) Filtering: Reads are subjected to quality score (Q-score) and length filtering via NanoFilt v2.8.0 (De Coster *et al.* 2018) to eliminate low-quality and chimeric sequences. (iii) Demultiplexing: Adapter removal and sample demultiplexing are performed using Porechop v0.2.4 (https://github.com/rrwick/Porechop) to segregate barcoded samples. (iv) Quality control: Sequencing statistics and quality metrics are visualized using NanoPlot v1.46.2 (De Coster and Rademakers 2023) to facilitate performance assessment. (v) Taxonomy: Taxonomic assignment is conducted using Emu 3.5.1 (Curry *et al.* 2022), an efficient classifier optimized for 16S full-length reads. To accommodate diverse research needs, microbiONT provides a multiple selection of pre-configured reference databases derived from various authoritative sources, covering both 16S and 18S rRNA genes. For 16S analysis, the platform includes both the default Emu database (accessed May 2023) and the NCBI 16S gene database (accessed May 2025). Furthermore, microbiONT incorporates two 18S rRNA gene databases targeting invertebrates and broader microbial groups (spanning Chromista, Plantae, and Protozoa), which were sourced from the MetaZooGene Barcode Atlas and Database (MZGdb, accessed January 2023) (Bucklin *et al.* 2021).

While microbiONT is equipped with an 18S rRNA gene database to facilitate eukaryotic profiling, we emphasize that the 18S analytical pipeline has not been rigorously benchmarked for quantitative accuracy in this study. The primary limitation stems from the extreme scarcity of standardized eukaryotic mock communities and publicly available datasets with established ground truths. Furthermore, quantitative 18S analysis faces intrinsic limitations: extreme intra- and inter-species gene copy number variations among microeukaryotes (Gong and Marchetti 2019), and the relative scarcity of curated eukaryotic reference databases compared to prokaryotic ones (Guillou *et al.* 2013, Bucklin *et al.* 2021). Consequently, we recommend that users currently utilize the 18S module primarily as an exploratory tool for qualitative community assessment, and interpret the resulting relative abundance metrics with appropriate caution.

This allows users to select the most appropriate reference dataset based on their specific biological context. (vi) Format conversion: Specialized Python scripts automatically convert taxonomy results into TSV abundance/taxonomy tables and Newick phylogenetic trees compatible with MicrobiomeAnalyst (Lu *et al.* 2023), as well as appropriately formatted count tables and representative FASTA sequences for functional prediction tools including PICRUSt2 (Douglas *et al.* 2020) and FAPROTAX (Louca *et al.* 2016).

### 2.3 User interface and pipeline flexibility

To complement the standardized workflow, microbiONT offers extensive operational flexibility through a user-friendly Streamlit interface. The GUI is structurally organized with a sidebar for module selection and a comprehensive main panel for execution monitoring, where real-time processing logs provide immediate feedback on workflow status while simultaneously hosting the interactive AI chat interface (Supplementary Fig. S1, available at supplementary material  *Bioinformatics Advances* online). In addition to the standardized One-Click workflow (Supplementary Fig. S2A, available at supplementary material  *Bioinformatics Advances* online), users can customize parameters for each processing step (i–vi) directly through the interface, allowing each module to be operated independently (Supplementary Fig. S2B, available at supplementary material  *Bioinformatics Advances* online). Furthermore, the platform features a Custom Pipeline mode for assembling tailored workflows (Supplementary Fig. S2C and D, available at supplementary material  *Bioinformatics Advances* online). For advanced requirements, an integrated command-line interface allows users to directly modify and execute raw commands (Supplementary Fig. S2E, available at supplementary material  *Bioinformatics Advances* online). Additionally, users can submit analysis requests using natural language; the AI assistant generates responses and executable commands, assisting users with parameter explanations and troubleshooting alongside their active analysis tasks (Supplementary Fig. S3, available at supplementary material  *Bioinformatics Advances* online).

### 2.4 AI integration and system deployment

microbiONT incorporates a local Large Language model (LLM), Llama 3.1 (https://ai.meta.com/blog/meta-llama-3/), through the Ollama framework (https://github.com/ollama/ollama). To optimize performance for specific analytical tasks, the model is configured using a custom Modelfile tailored to microbiONT architecture. This module interprets natural language queries to offer technical explanations of analysis parameters and bioinformatics guidance, functioning as an interactive reference system.

To evaluate the reliability of the AI assistant, we conducted a rigorous benchmark using 30 queries across six core workflow categories (e.g. basecalling, quality control, taxonomy). Following standard LLM evaluation methodologies, accuracy was measured using the pass@k metric (Chen, et al., 2021), assessing both the strict correctness (accurate parameter selection) and completeness (executable syntax) of the generated commands. Validation results indicate that the model achieves an initial accuracy (pass@1) of 71.3% (Supplementary Table S3, available at supplementary material  *Bioinformatics Advances* online). Notably, the use of iterative prompting (pass@5), increases accuracy to over 96%, demonstrating the system’s robust capability to generate correct responses and executable commands even from simplified or variable user prompts (See Supplementary File 1 and Supplementary Table S3, available at supplementary material  *Bioinformatics Advances* online). To further facilitate practical application, we provide a curated set of verified, pass@1 prompt templates derived from this benchmark (Supplementary File 2, available at supplementary material  *Bioinformatics Advances* online). These templates have been optimized for common analytical tasks, allowing users to directly copy or minimally adapt them (e.g. modifying input filenames) for immediate and highly accurate AI execution.

To address challenges related to software compatibility and environment configuration, microbiONT employs a user-friendly, shell script-based installation mechanism designed for simplicity. The deployment process is streamlined into two steps: users execute the install.sh script to automatically establish the required environment and dependencies, and subsequently launch the platform via the runtime script (./run.sh), which initiates the web-based interface.

### 2.5 Criticality and mitigation of AI-generated errors

Although the customized Modelfile significantly improves prompt adherence, the inherently generative nature of LLMs leaves room for functional errors. While the overall success rate remains high, we have detailed the potential error patterns that may arise. To assess the consequences of these errors and justify the system’s user interface design, the observed AI failures were categorized by their potential impact on data integrity (Supplementary Table S4, available at supplementary material  *Bioinformatics Advances* online). This evaluation distinguishes between “loud” and “silent” failures. Loud errors, such as the generation of non-existent tool parameters (Parameter Hallucinations), represent “safe failures”; the terminal immediately rejects the invalid commands, thereby precluding data corruption. Conversely, a more substantial risk arises from silent errors, specifically defined as Logical Misinterpretations. In such cases, the AI produces syntactically valid but methodologically flawed commands. If executed without manual verification, these commands proceed without system warnings, potentially causing undetected over- or under-correction of reads and subsequent taxonomic misclassifications. To address the risk of silent analytical failures, microbiONT incorporates an AI-assisted, human-in-the-loop framework. By requiring explicit user validation of the generated bash commands prior to execution, the platform ensures that researchers retain continuous technical oversight, preventing logical inaccuracies from propagating into downstream biological interpretations.

## 3 Results

### 3.1 Cross-platform performance and resource efficiency

To validate the system’s stability and versatility, we processed a raw Pod5 dataset containing 1 million reads on both native Linux environments and Windows systems utilizing the Windows Subsystem for Linux 2 (WSL2). Detailed hardware specifications for the testing environments are provided in Table S1, available at supplementary material  *Bioinformatics Advances* online.

Resource monitoring analysis identified basecalling and AI inference as the primary consumers of GPU memory. Notably, while Machine A exhibited significantly higher peak VRAM usage during basecalling compared to the AI inference step, VRAM consumption was comparable between these two stages across the other test configurations (Table S2, available at supplementary material  *Bioinformatics Advances* online). These findings allow us to conclude that integrating the AI module does not impose hardware requirements exceeding those already mandated by standard Nanopore basecalling. Benchmarking confirms that the workflow efficiently processes standard MinION amplicon runs on consumer-grade laptops equipped with NVIDIA GPUs (a minimum of 6 GB VRAM is required, while 8 GB or more is recommended for optimal AI inference).

### 3.2 Benchmarking taxonomic accuracy using mock communities

To rigorously evaluate taxonomic classification accuracy, we analyzed three independent DNA samples extracted from the ZymoBIOMICS® Microbial Community Standard (D6300). We compared the performance of microbiONT v1.0.0 (utilizing the Emu-based custom workflow) against the widely used EPI2ME Desktop v5.3.0 platform (utilizing the Minimap2-based workflow) by calculating the coefficient of determination (*R*^2^) and the root mean square error (RMSE) between the observed and expected relative abundances of the bacterial genera.

Benchmarking results revealed a consistent performance advantage for microbiONT. Across three replicates, microbiONT demonstrated higher classification precision, achieving *R*^2^ values from 0.76 to 0.82 and RMSE values ranging from 2.41 to 2.81. In contrast, the EPI2ME workflow exhibited significantly higher deviation, with poor model fit with *R*^2^ values strictly below 0.5 (0.35–0.40) and RMSE values ranging between 4.42 and 4.63 (Fig. S4, available at supplementary material  *Bioinformatics Advances* online). A paired Student’s *t*-test confirmed that microbiONT’s improvements in both predictive error (RMSE) and goodness of fit (*R*^2^) are statistically significant (*P *< 0.05). A detailed genus-level analysis identified the primary source of this discrepancy. The EPI2ME workflow showed substantial misclassification, particularly within the *Escherichia* genus and an inflated assignment of reads to the others category. This tendency to misclassify reads into non-target groups suggests a limitation in the standard EPI2ME classification strategy for this specific mock community. Conversely, microbiONT’s integrated workflow, which leverages the Emu algorithm alongside customized parameter optimization, effectively minimized these off-target assignments, resulting in a community profile that more closely mirrored the theoretical composition.

While full-length 16S/18S sequencing theoretically provides species-level resolution, our evaluation reveals that classification accuracy at this taxonomic rank is highly volatile and heavily dependent on the composition of the reference database. As demonstrated in our supplementary evaluation (Table S5, available at supplementary material  *Bioinformatics Advances* online), relying exclusively on standard public databases (e.g. unmodified NCBI) results in severe taxonomic deviations, including extreme false-negative rates and inflated non-target assignments. Although microbiONT can achieve accurate species-level profiling when supplemented with specific reference sequences (Fig. S5, available at supplementary material  *Bioinformatics Advances* online), standard databases frequently lack the necessary completeness to resolve taxa with high sequence homology reliably. Therefore, given the significant discrepancies introduced by database variations, we strongly recommend utilizing the genus level as the primary and most robust basis for standard taxonomic interpretation.

## 4 Conclusion

The microbiONT package represents a paradigm shift towards accessible, educational, and privacy-centric bioinformatics. By synergizing established CLI tools with an intuitive graphical interface and a local AI module, the platform effectively dismantles the technical barriers associated with Nanopore data analysis. This design empowers microbiologists to not only perform comprehensive 16S/18S analysis independently but also to cultivate bioinformatics expertise through AI-assisted guidance. Crucially, this accessibility does not come at the cost of precision; our benchmarking demonstrates that microbiONT yields a more accurate taxonomic profile than the standard EPI2ME workflow, characterized by a marked reduction in unclassified reads and enhanced genus-level resolution. Ultimately, driven by AI-guided support, local processing, and analytical rigor, microbiONT transforms 16S/18S data analysis from a specialized bottleneck into a convenient, routine practice for every laboratory.

## Supplementary Material

vbag229_Supplementary_Data

## Data Availability

The data underlying this article are available in *microbiONT* at https://github.com/MBEBlab/microbiONT.

## References

[vbag229-B1] Bucklin A , Peijnenburg KTCA, Kosobokova KN et al Toward a global reference database of COI barcodes for marine zooplankton. Mar Biol 2021;168:78.

[vbag229-B2] Chen M , Tworek J, Jun H et al Evaluating large language models trained on code. arXiv preprint arXiv:2107.03374 2021, preprint: not peer reviewed.

[vbag229-B3] Curry KD , Wang Q, Nute MG et al Emu: species-level microbial community profiling of full-length 16S rRNA Oxford Nanopore sequencing data. Nat Methods 2022;19:845–53. 35773532 10.1038/s41592-022-01520-4 PMC9939874

[vbag229-B4] De Coster W , D’Hert S, Schultz DT et al NanoPack: visualizing and processing long-read sequencing data. Bioinformatics 2018;34:2666–9. 29547981 10.1093/bioinformatics/bty149 PMC6061794

[vbag229-B5] De Coster W , Rademakers R. NanoPack2: population-scale evaluation of long-read sequencing data. Bioinformatics 2023;39:btad311. 37171891 10.1093/bioinformatics/btad311 PMC10196664

[vbag229-B6] Douglas GM , Maffei VJ, Zaneveld JR et al PICRUSt2 for prediction of metagenome functions. Nat Biotechnol 2020;38:685–8. 32483366 10.1038/s41587-020-0548-6 PMC7365738

[vbag229-B7] Gong W , Marchetti A. Estimation of 18S gene copy number in marine eukaryotic plankton using a next-generation sequencing approach. Front Mar Sci 2019;6:219.

[vbag229-B8] Guillou L , Bachar D, Audic S et al The Protist Ribosomal Reference database (PR2): a catalog of unicellular eukaryote small sub-unit rRNA sequences with curated taxonomy. Nucleic Acids Res 2013;41:D597–604. 23193267 10.1093/nar/gks1160 PMC3531120

[vbag229-B9] Li H , Handsaker B, Wysoker A et al The sequence alignment/map format and SAMtools. Bioinformatics 2009;25:2078–9. 19505943 10.1093/bioinformatics/btp352 PMC2723002

[vbag229-B10] Louca S , Parfrey LW, Doebeli M. Decoupling function and taxonomy in the global ocean microbiome. Science (1979) 2016;353:1272–7. 10.1126/science.aaf4507 27634532

[vbag229-B11] Lu Y , Zhou G, Ewald J et al MicrobiomeAnalyst 2.0: comprehensive statistical, functional and integrative analysis of microbiome data. Nucleic Acids Res 2023;51:W310–8. 37166960 10.1093/nar/gkad407 PMC10320150

[vbag229-B12] Rodríguez-Pérez H , Ciuffreda L, Flores C. NanoCLUST: a species-level analysis of 16S rRNA nanopore sequencing data. Bioinformatics 2021;37:1600–1. 33079990 10.1093/bioinformatics/btaa900

[vbag229-B13] Santos A , van Aerle R, Barrientos L et al Computational methods for 16S metabarcoding studies using Nanopore sequencing data. Comput Struct Biotechnol J 2020;18:296–305. 32071706 10.1016/j.csbj.2020.01.005 PMC7013242

[vbag229-B14] Zorz J , Li C, Chakraborty A et al SituSeq: an offline protocol for rapid and remote Nanopore 16S rRNA amplicon sequence analysis. ISME Commun 2023;3:33. 37081077 10.1038/s43705-023-00239-3 PMC10119094

