# Supplementary material for: microbiONT: an AI-assisted, privacy-focused platform for local Nanopore 16S and 18S amplicon analysis
Source: Bioinform Adv. 2026 Aug 12;6(1):vbag229. doi: 10.1093/bioadv/vbag229 (PMC13499628; doi:10.1093/bioadv/vbag229)
Supplement: Supplementary file 1 [file vbag229_supplementary_data.zip › revise2_supplementary_data.docx]

**Supplementary data**

**microbiONT: an AI-assisted, privacy-focused platform for local Nanopore 16S and 18S amplicon analysis**

**Che-Chun Chen^1,2,3^, Hsin-Yun Lu^1^, and Ying-Ning Ho^1,3,4*^**

1 Institute of Marine Biology, National Taiwan Ocean University, Keelung, Taiwan

2 Department of Aquaculture, National Taiwan Ocean University, Keelung, Taiwan

3 Taiwan Oceans Genome Center, National Taiwan Ocean University, Keelung, Taiwan

4 Center of Excellence for the Oceans, National Taiwan Ocean University, Keelung, Taiwan

*Correspondence:

Ying-Ning Ho

ynho@email.ntou.edu.tw

**Declaration of Generative AI and AI-assisted Technologies:**

During the preparation of this work, the authors utilized Google Gemini to improve the readability and grammatical accuracy of the manuscript. The tool was also employed to assist in code generation, debugging, and the identification of potential anomalies within the codebase. The authors reviewed and edited the content as needed and take full responsibility for the content of the publication.

This supplementary file includes the following content:

**Supplementary Table S1**. Hardware specifications of the computing systems used for performance benchmarking.

**Supplementary Table S2**. Computational resource utilization profile across different steps of the microbiONT workflow and AI inference.

**Supplementary Table S3.** Accuracy of AI-generated commands evaluated using the pass@k metric.

**Supplementary Table S4.** Categorization and criticality of AI-generated command outcomes and errors.

**Supplementary Table S5.** Impact of database composition on species-level taxonomic classification accuracy.

**Supplementary Figure S1**. Overview of the microbiONT Graphical User Interface.

**Supplementary Figure S2**. Demonstration of modular flexibility and advanced control features.

**Supplementary Figure S3**. AI Copilot interaction and natural language processing capabilities.

**Supplementary Figure S4**: Taxonomic classification accuracy of microbiONT versus EPI2ME using the mock community.

**Supplementary Figure S5.** Species-level taxonomic classification accuracy of microbiONT utilizing a customized reference database.

**Supplementary Methods**

**Performance Benchmarking Setup**

To evaluate the system's performance across different environments, we utilized four distinct computing systems, comprising two desktop workstations running Linux and two laptops operating on Windows. Detailed hardware specifications for each system are provided in Supplementary Table S1. Performance benchmarking was conducted using a raw Pod5 file containing 1,059,785 full-length 16S rRNA sequencing reads. We monitored three performance metrics including total execution time (seconds), peak system memory usage (RAM in GB), and peak video memory usage (VRAM in MiB). The evaluation covered the complete analysis workflow, encompassing basecalling, quality filtering, demultiplexing, quality control, taxonomic classification, and format conversion. Additionally, we assessed the computational load of the AI module by querying the assistant with a set of 100 microbiONT-related questions (average query length: 49.6 characters). All tools within the microbiONT pipeline were executed using their default parameters. The resulting resource utilization profiles are summarized in Supplementary Table S2, with detailed procedures and commands described below.

\# Step 1: Basecalling

"dorado basecaller sup '$POD5_DIR' > calls.bam" & "samtools fastq calls.bam > calls.fastq"

\# Step 2: Filtering

"NanoFilt -q 15 -l 1450 --maxlength 1650 --readtype 1D calls.fastq > filtered.fastq"

\# Step 3: Demultiplex

"porechop -i filtered.fastq -b porechop_out --barcode_threshold 85 --barcode_diff 1"

\# Step 4: Quality Control

"NanoPlot --fastq porechop_out/*.fastq -o qc_report_final --legacy hex dot"

\# Step 5: Taxonomy

CMD_EMU="mkdir -p taxonomy_out && \

for f in porechop_out/*.fastq; do \

if [ -f \"\$f\" ]; then \

emu abundance --type map-ont --db '$EMU_DB' --keep-counts \"\$f\" --output-dir taxonomy_out; \

fi; \

done && \

emu combine-outputs taxonomy_out tax_id --counts"

\# Step 6: Formal conversion

"python3 '$POST_SCRIPT' -i taxonomy_out -o conversion_out -d '$EMU_DB' --ma"

**Mock Community Sample Preparation and Sequencing**

To evaluate taxonomic accuracy, the ZymoBIOMICS^®^ Microbial Community Standard D6300 (Zymo Research, USA) was selected as the reference material. Genomic DNA was extracted using the DNeasy PowerSoil Pro Kit (QIAGEN, Germany) following the manufacturer’s instructions. The V1-V9 regions of the 16S rRNA gene were amplified using universal primers 27F (5’-AGAGTTTGATCCTGGCTCAG-3’) and 1492R (5’-GGTTACCTTGTTACGACTT-3’) (Klindworth, et al., 2013), incorporating unique barcodes for multiplexing. PCR amplification involved an initial denaturation at 95°C for 5 min, followed by 30 cycles of denaturation (95°C, 30 s), annealing (50°C, 30 s), and extension (72°C, 90 s), concluding with a final extension at 72°C for 5 min. Amplicons were purified with AMPure XP beads (Beckman Coulter, USA) and quantified using a Qubit™ 4 Fluorometer (Thermo Fisher Scientific, USA). Sequencing libraries were prepared according to the Ligation Sequencing Kit V14 protocol (SQK-LSK114; Oxford Nanopore Technologies, UK) and sequenced on R10.4.1 MinION flow cells for approximately 96 hours.

**Bioinformatics Benchmarking and Database Configuration**

We benchmarked the classification performance of microbiONT v1.0.0 against the standard EPI2ME Desktop v5.3.0 workflow by calculating the root mean squared error (RMSE) and R^2^ between the observed and expected relative abundances of the seven bacterial genera in the mock community Our methodology was adapted from the approach described by Urban et al.(Urban, et al., 2021). To ensure reliability, we performed three independent random subsamplings of 100,000 reads from the dataset. The outcomes remained consistent across all three replicates (average RMSE = 2.73 ± 0.06), confirming that our evaluation provides a robust and accurate measure of each classifier’s performance.

For the EPI2ME 16S rRNA workflow, the minimap2 aligner was employed, whereas microbiONT utilized the Emu algorithm (Curry, et al., 2022). Both EPI2ME and microbiONT 16S workflow were executed using its same EPI2ME default database configuration (https://ont-exd-int-s3-euwst1-epi2me-labs.s3.amazonaws.com/wf-metagenomics/ncbi_16s_18s/ncbi_targeted_loci_16s_18s.fna). Additionally, microbiONT includes pre-configured databases to facilitate broad usage, including 18S rRNA sequences for invertebrates and Microbes & Protists (including Chromista, Plantae, and Protozoa) sourced from the MetaZooGene Barcode Atlas and Database (MZGdb, accessed January 2023)(Bucklin, et al., 2021). Furthermore, microbiONT supports the integration of custom user-defined databases in an Emu-compatible format; detailed instructions for constructing compatible databases are available at https://github.com/treangenlab/emu.

**Supplementary Results**

**Cross-Platform Performance and Resource Efficiency**

To validate cross-platform stability and characterize resource efficiency, we benchmarked microbiONT using a standardized dataset of 1 million raw reads (Pod5 format) across four distinct hardware configurations (Machines A-D; see Supplementary Table S1) utilizing both native Linux and Windows Subsystem for Linux 2 (WSL2) environments. Detailed resource monitoring identified basecalling (Dorado) and AI inference (Llama 3.1) as the primary consumers of GPU memory; however, utilization patterns varied by hardware capability (Supplementary Table S2). The high-performance Machine A exhibited high VRAM usage for basecalling (16.6 GB), which was significantly higher than that of AI inference (8.1 GB). In contrast, Machines B, C, and D displayed convergent patterns, with comparable peak VRAM consumption across both stages. Performance analysis for the AI copilot (average query length: 49.6 characters) revealed that Machines A and B achieved optimal responsiveness (~3.5 s inference time; ~8 GB VRAM), while Machine C exhibited intermediate latency (~7.6 s); notably, Machine D, operating under hardware constraints with approximately 5.4 GB VRAM usage, experienced significantly increased latency (>30 s) likely due to memory saturation. Collectively, these findings demonstrate that integrating the local AI module does not impose hardware requirements exceeding those already mandated by standard Nanopore basecalling, confirming that while a minimum of 6 GB VRAM is viable for functional execution, a specification of 8 GB or higher is recommended to ensure optimal real-time interaction.

**Benchmarking Taxonomic Accuracy using Mock Communities**

To evaluate the taxonomic classification performance of microbiONT v1.0.0 and EPI2ME Desktop v5.3.0, we analyzed sequencing data generated from the ZymoBIOMICS® Microbial Community Standard (D6300) using the V1–V9 primer pair (27F/1492R), with each test replicate comprising a raw dataset of 100,000 reads. Taxonomic profiles were generated using two distinct strategies: EPI2ME (utilizing Minimap2 alignments against its default database) and microbiONT (utilizing Emu alignments against the NCBI 16S gene database).

We first assessed the proportion of reads successfully assigned to the eight expected genera present in the mock community (ordered by theoretical abundance: *Limosilactobacillus*, *Bacillus*, *Staphylococcus*, *Listeria*, *Salmonella*, *Escherichia*, *Enterococcus*, and *Pseudomonas*). As illustrated in Supplementary Figure S4, microbiONT demonstrated superior classification sensitivity, with an average of 98.27% of classified reads aligned to the expected taxa. In contrast, EPI2ME assigned only 89.67% of reads to the expected genera, with a significant proportion categorized as Others (non-target taxa). Quantitative accuracy was assessed using the coefficient of determination (R^2^) and the Root Mean Square Error (RMSE) between observed and expected relative abundances. microbiONT consistently achieved higher R^2^ values (average R^2^ = 0.78) and lower RMSE values (average RMSE = 2.66) compared to EPI2ME (average R^2^ = 0.37; average RMSE = 4.53). A paired Student's t-test indicated that these differences in overall fit metrics (R^2^ and RMSE) are statistically significant (*p* < 0.05). These metrics indicate that the microbiONT workflow produced abundance profiles that were highly consistent across replicates and more representative of the theoretical composition.

While both platforms exhibited similar deviation trends (simultaneous over- or under-estimation) for most of the genera, a distinct divergence was observed in the classification of *Escherichia* and the Others category. Specifically, microbiONT overestimated *Escherichia* abundance by 4.37%, whereas EPI2ME underestimated it by 4.9%. Crucially, this underestimation in EPI2ME was accompanied by a 10.33% overrepresentation in the others category. Statistical analysis confirmed significant differences (*p* < 0.05) in the relative abundance quantification of *Bacillus*, *Escherichia*, and the Others category between the two pipelines.

These results suggest that the standard EPI2ME workflow lacks the resolution to accurately resolve certain 16S amplicons (particularly *Escherichia*), frequently misclassifying them into the others group. Conversely, microbiONT's integration of the Emu algorithm and a comprehensive NCBI database effectively retrieves these reads, minimizing false-negative classifications and providing a more accurate representation of the microbial community structure.

**Volatility of Species-Level Profiling and Database Dependency**

To empirically demonstrate the volatility and database dependency of species-level profiling, we repeated the classification analysis using the default, unmodified NCBI 16S reference database (Supplementary Table S5). Reliance on the standard database resulted in substantial taxonomic misclassification. Specifically, the proportion of reads assigned to the non-target Others category increased dramatically, averaging 63.28% (range: 62.63–63.68%). Correspondingly, several target taxa exhibited pronounced false-negative rates. For example, Salmonella enterica was not detected in any replicate (0.00% across all samples). In addition, *Staphylococcus aureus*, *Pseudomonas aeruginosa*, and *Limosilactobacillus fermentum* were markedly underestimated, with average classification rates below 0.2% (range: 0.01–0.17%). These findings demonstrate that species-level assignments are highly sensitive to the choice of reference database and can lead to substantial quantitative discrepancies. Given this inherent instability and the associated risk of erroneous species-level profiling, we conclude that genus-level classification remains the most robust and reliable taxonomic resolution for microbiome analyses.

**Supplementary Tables**

**Supplementary Table S1. Hardware specifications of the computing systems used for performance benchmarking.** The benchmarking environment consisted of four distinct setups: two desktop workstations running native Linux and two consumer-grade laptops running Windows (via WSL2). Specifications include operating system, CPU model, CPU cores, CPU threads, total RAM, GPU model, and GPU VRAM capacity.

| **Hardware Component** | **Machine A (Native Linux Desktop)** | **Machine B (Native Linux Desktop)** | **Machine C (Laptop / WSL2)** | **Machine D (Laptop / WSL2)** |
| --- | --- | --- | --- | --- |
| Operating System | Ubuntu 22.04.5 LTS | Ubuntu 22.04.5 LTS | Windows 11 Home (25H2) via WSL2 (Ubuntu 24.04.3 LTS) | Windows 11 Home (25H2) via WSL2 (Ubuntu 24.04.3 LTS) |
| CPU | AMD Ryzen Threadripper 3960X Processor @3.8GHz | AMD Ryzen 9 3950X Processor @ 3.5GHz | AMD Ryze AI 9 HX 370 Processor @ 2.0GHz | AMD Ryzen 5800H @3.2GHz |
| Core | 24 | 16 | 12 | 8 |
| CPUs | 48 | 32 | 24 | 16 |
| RAM | 256 GB DDR4 3200MHz | 128 GB DDR4 3200MHz | 64 GB LPDDR5X 8000MHz | 16 GB DDR4 3200MHz |
| GPU | NVIDIA GeForce RTX 4090 | NVIDIA GeForce RTX 3080 | NVIDIA GeForce RTX 5070 Ti Laptop GPU | NVIDIA GeForce RTX 3060 Laptop GPU |
| GPU Memory (VRAM) | 24 GB GDDR6X | 12 GB GDDR6X | 12 GB GDDR7 | 6 GB GDDR6 |

**Supplementary Table S2. Computational resource utilization profile across different steps of the microbiONT workflow and AI inference.** Performance metrics, including execution time (seconds), peak system memory (RAM), and peak video memory (VRAM) were recorded while processing a dataset of 1,059,785 full-length 16S reads. The evaluation encompasses all primary pipeline stages (basecalling, filtering, demultiplexing, quality control, taxonomy, and format conversion) as well as the inference load for the AI assistant (answering 100 queries with an average length of 49.6 characters). All analyses were conducted in accordance with the default parameters established by microbiONT.

| **Machine** | **Step** | **Time_Sec** | **Peak_RAM_GB** | **Peak_VRAM_MiB** |
| --- | --- | --- | --- | --- |
| A | 1_Basecalling | 2617 | 25.92 | 16590 |
|  | 2_Filtering | 155 | 0.12 | 1166 |
|  | 3_Demultiplexing | 5496 | 8.57 | 1222 |
|  | 4_Quality control | 39.73 | 0.28 | 1222 |
|  | 5_Taxonomy | 14707 | 0.43 | 1232 |
|  | 6_Format conversion | 0.43 | 0.11 | 1162 |
|  | AI Inference | 3.4187 | 6.51 | 8061.5 |
| B | 1_Basecalling | 7195 | 25.8 | 6261 |
|  | 2_Filtering | 130 | 0.12 | 626 |
|  | 3_Demultiplexing | 4596 | 8.59 | 772 |
|  | 4_Quality control | 44 | 0.31 | 763 |
|  | 5_Taxonomy | 15629 | 0.4 | 767 |
|  | 6_Format conversion | <0.0001 | 0.12 | 695 |
|  | AI Inference | 3.6751 | 5.49 | 7277.7 |
| C | 1_Basecalling | 8362 | 25.9 | 8645 |
|  | 2_Filtering | 90 | 0.12 | 1901 |
|  | 3_Demultiplexing | 19822 | 8.58 | 1911 |
|  | 4_Quality control | 24 | 0.29 | 1901 |
|  | 5_Taxonomy | 9995 | 0.41 | 1901 |
|  | 6_Format conversion | <0.0001 | 0.11 | 1901 |
|  | AI Inference | 7.6307 | 1.79 | 7801.1 |
| D | 1_Basecalling | 19677 | 6.79 | 4937 |
|  | 2_Filtering | 179 | 0.12 | 1075 |
|  | 3_Demultiplexing | 15086 | 6.96 | 1057 |
|  | 4_Quality control | 57 | 0.3 | 941 |
|  | 5_Taxonomy | 19195 | 0.42 | 1009 |
|  | 6_Format conversion | 1 | 0.11 | 1004 |
|  | AI Inference | 33.3625 | 1.43 | 5388.7 |

**Supplementary Table S3.** **Accuracy of AI-generated commands evaluated using the pass@k metric.** The table presents the pass@k (k=1 to 5) evaluation results for the customized LLM. The assessment encompasses 30 natural language queries across six primary functional categories corresponding to the microbiONT workflow: basecalling, filtering, demultiplexing, quality control, taxonomy, and format conversion. The data includes the individual accuracy percentages for each category across 1 to 5 generated attempts, as well as the overall average accuracy metrics for the entire benchmark dataset.

| Prompt Category | pass@1 | pass@2 | pass@3 | pass@4 | pass@5 |
| --- | --- | --- | --- | --- | --- |
| 1. Basecalling | 100.0% | 100.0% | 100.0% | 100.0% | 100.0% |
| 2. Filtering | 80.0% | 92.0% | 98.0% | 100.0% | 100.0% |
| 3. Demultiplexing | 84.0% | 88.0% | 92.0% | 96.0% | 100.0% |
| 4. Quality control | 40.0% | 64.0% | 80.0% | 92.0% | 100.0% |
| 5. Taxonomy | 52.0% | 62.0% | 70.0% | 76.0% | 80.0% |
| 6. Format conversion | 72.0% | 82.0% | 90.0% | 96.0% | 100.0% |
| Overall | 71.3% | 81.3% | 88.3% | 93.3% | 96.7% |

**Supplementary Table S4. Categorization and criticality of AI-generated command outcomes and errors.** This table classifies the AI command generation outcomes observed during the prompt adherence benchmarking into five distinct categories: Pass (Correct & Complete), Textual Description Error, Parameter Omission, Parameter Hallucination, and Logical Misinterpretation. Specifications for each category include the assigned criticality level (ranging from None to High), a description of the outcome or error mechanism, and a specific example derived from the benchmark testing.

| Error Type | Criticality | Description & Example |
| --- | --- | --- |
| Pass (Correct & Complete) | None | The generated command is perfectly executable, completely fulfills the user's constraints, and the accompanying text explanation is accurate. |
| Textual Description Error | No impact | The generated bash command is executable and functionally correct, but the accompanying natural language explanation contains inaccuracies.  **Example:** The AI generates the correct command NanoFilt -q 15, but the text mistakenly states, "This keeps reads with Q>10." |
| Parameter Omission | Low (loud failure) | The generated command is structurally valid but omits specific parameter configurations requested by the user or required by the tool.  **Example:** The user requested to convert Emu results for MicrobiomeAnalyst and PICRUSt2 compatibility, but the generated command omitted the required database file path argument. |
| Parameter Hallucination | Low to Moderate (loud failure) | The LLM fabricates non-existent tool functions, flags, or parameters.  **Example:** The user requested to keep read lengths between 1000 and 1800 bp, but the AI generated an invalid command using NanoFilt -l 1000 -L 1800, where -L is a non-existent parameter. |
| Logical Misinterpretation | High (silent failure) | The command is syntactically valid but performs an incorrect operation due to a misunderstanding of the user's intent.  **Example:** The user requested to keep reads with a maximum length of 2000 bp, but the AI incorrectly restricted the reads to exactly 2000 bp by generating NanoFilt -l 2000 --maxlength 2000. |

**Supplementary Table S5. Impact of database composition on species-level taxonomic classification accuracy.** The table presents the species-level relative abundance profiles for the eight expected target bacteria and the non-target Others group across three independent replicates. Classifications were evaluated under two distinct reference database conditions: a customized database (NCBI DB with MOCK) supplemented with specific reference sequences from the ZymoBIOMICS Microbial Community Standard, and the default, unmodified standard reference database (NCBI DB).

|  | Expected | NCBI DB with MOCK | | | NCBI DB | | |
| --- | --- | --- | --- | --- | --- | --- | --- |
| *Limosilactobacillus fermentum* | 18.4 | 13.8 | 13.0 | 13.4 | 0.10 | 0.17 | 0.17 |
| *Bacillus subtilis* | 17.4 | 17.2 | 16.4 | 16.3 | 8.18 | 8.05 | 8.13 |
| *Staphylococcus aureus* | 15.5 | 14.3 | 14.1 | 13.6 | 0.01 | 0.03 | 0.07 |
| *Listeria monocytogenes* | 14.1 | 13.2 | 12.8 | 12.4 | 13.67 | 12.85 | 13.31 |
| *Salmonella enterica* | 10.4 | 13.2 | 14.1 | 14.2 | 0.00 | 0.00 | 0.00 |
| *Escherichia coli* | 10.1 | 13.4 | 14.1 | 14.2 | 1.95 | 2.13 | 2.26 |
| *Enterococcus faecalis* | 9.9 | 8.2 | 8.3 | 8.3 | 12.31 | 13.13 | 13.32 |
| *Pseudomonas aeruginosa* | 4.2 | 1.8 | 2.1 | 2.2 | 0.10 | 0.12 | 0.11 |
| Others | 0.0 | 4.9 | 5.2 | 5.5 | 63.68 | 63.53 | 62.63 |

**Supplementary Figures**

**Figure S1. Overview of the microbiONT Graphical User Interface.**

The microbiONT interface is organized into two main sections: (A) Main Menu; (B) AI Copilot Interface, users can submit analysis requests using natural language, this section include logs the conversation between the user and the AI agents.

**Figure S2. Demonstration of modular flexibility and advanced control features.** (A) The Standardized SOP interface for routine One-Click analysis. (B) Configuration panels allow for independent execution of specific modules (e.g., Basecalling) with customizable parameters. (C-D) The Custom Pipeline interface enables users to assemble and execute tailored workflows. (E) The integrated command-line interface (CLI) enables advanced users to execute raw bioinformatics commands directly within the GUI.

**Figure S3. AI Copilot interaction and natural language processing capabilities**. (A) User input queries expressed in natural language; (B) The AI's descriptive response; (C) The corresponding executable command syntax generated by the model. Example of the AI assistant interpreting natural language queries to generate accurate bioinformatics commands (e.g., for filtering file), explaining parameters, and providing troubleshooting guidance.

**Figure S4. Taxonomic classification accuracy of microbiONT versus EPI2ME using the mock community.** Comparison of observed versus expected relative abundances for the eight expected genera and the non-target others group. The figure presents stacked bar charts visualizing deviation from theoretical values (left), detailed abundance percentages (right), coefficient of determination (R^2^), and Root Mean Square Error (RMSE) scores for each replicate (bottom). Classifications were performed using EPI2ME default NCBI 16S and 18S rRNA gene database. microbiONT demonstrated superior fidelity, achieving significantly higher R^2^(0.76 - 0.82) and lower RMSE values (2.41 -2.81) compared to EPI2ME (R^2^: 0.35-0.40; RMSE: 4.42 - 4.63). Asterisks (*) denote statistically significant differences between microbiONT and EPI2ME (*p* < 0.05), as determined by a paired Student's t-test.

**Figure S5. Species-level taxonomic classification accuracy of microbiONT utilizing a customized reference database.** The figure presents the analysis of observed versus expected relative abundances at the species level for the eight target bacteria and the non-target Others group. Stacked bar charts (left) visualize the community profile and its deviation from the theoretical baseline, while the accompanying table (right) details the specific abundance percentages across three independent test replicates. Classifications were executed using the microbiONT pipeline coupled with a customized NCBI 16S database, which was specifically supplemented with reference sequences from the ZymoBIOMICS Microbial Community Standard. The coefficient of determination (R^2^) and Root Mean Square Error (RMSE) scores, evaluating the quantitative fidelity and goodness of fit of the species-level assignments for each replicate, are provided at the bottom.

**References**

Bucklin, A.*, et al.* Toward a global reference database of COI barcodes for marine zooplankton. *Marine Biology* 2021;168(6):78.

Curry, K.D.*, et al.* Emu: species-level microbial community profiling of full-length 16S rRNA Oxford Nanopore sequencing data. *Nature methods* 2022;19(7):845–853.

Klindworth, A.*, et al.* Evaluation of general 16S ribosomal RNA gene PCR primers for classical and next-generation sequencing-based diversity studies. *Nucleic acids research* 2013;41(1):e1–e1.

Urban, L.*, et al.* Freshwater monitoring by nanopore sequencing. *elife* 2021;10:e61504.
