# Supplementary material for: microbiONT: an AI-assisted, privacy-focused platform for local Nanopore 16S and 18S amplicon analysis
Source: Bioinform Adv. 2026 Aug 12;6(1):vbag229. doi: 10.1093/bioadv/vbag229 (PMC13499628; doi:10.1093/bioadv/vbag229)
Supplement: Supplementary file 1 [file vbag229_supplementary_data.zip › revise2_supplementary_file_2.docx]

**Supplementary File 2**

**microbiONT: an AI-assistant, privacy-focused platform for local Nanopore amplicon analysis**

**Che-Chun Chen^1,2,3^, Hsin-Yun Lu^1^, and Ying-Ning Ho^1,3,4*^**

1 Institute of Marine Biology, National Taiwan Ocean University, Keelung, Taiwan

2 Department of Aquaculture, National Taiwan Ocean University, Keelung, Taiwan

3 Taiwan Oceans Genome Center, National Taiwan Ocean University, Keelung, Taiwan

4 Center of Excellence for the Oceans, National Taiwan Ocean University, Keelung, Taiwan

*Correspondence:

Ying-Ning Ho

ynho@mail.ntou.edu.tw

**Supplementary File 2:** Recommended quick-start prompts for optimal AI assistant performance

This supplementary file serves as a quick-start guide for the microbiONT AI copilot. It features a curated selection of prompt templates extracted from our comprehensive benchmarking dataset (Supplementary File 1). All included prompts achieved a **pass@1 accuracy**, generating strictly correct and fully executable commands on the first attempt. To maximize real-world utility, we slightly modified specific parameters within these verified templates to reflect the most frequently utilized functions. Users are highly encouraged to copy or adapt these prompts (e.g., by updating input filenames) for efficient and accurate AI interaction.

- **Basecalling (Dorado & Samtools)**

1. **How do I run basecalling on my pod5 files using the SUP model?**

**AI response:**

To run basecalling on your `.pod5` files using the SUP model, follow these steps:

Make sure you have the correct input: Ensure that the Input field in the settings is set to the path of your .pod5 file(s). You can browse for the file or enter the path manually.

Select the SUP model: In the basecalling settings, select the "SUP" model from the dropdown menu.

Run Dorado: Click on the "Run" button to start the basecalling process using the SUP model.

Here's a sample command that you can use in the terminal:

This will run Dorado with the SUP model on your .pod5 file and output the results in BAM format. You can then convert the BAM file to FASTQ using samtools.

Note: Make sure you have the correct permissions and that the input file is accessible by the system.

If you encounter any issues or have further questions, feel free to ask!

**Terminal Request:**

dorado basecaller sup pod5/ > calls.bam && samtools fastq calls.bam > calls.fastq

2. **Run basecalling and save the output to 'calls.bam'.**

**AI response:**

To run basecalling, I'll need to use Dorado. Since your input is not set, I'll assume you want to use the default input file.

Here's the command:

This will run Dorado on the pod5 file and save the output to a BAM file named calls.bam.

Please note that this will overwrite any existing file with the same name. If you want to append to an existing file, please let me know.

Also, if you have any specific settings or parameters for Dorado, feel free to share them, and I'll update the command accordingly.

Once you're ready, just click on the "Run" button to execute the command!

**Terminal Request:**

dorado basecaller sup pod5/ > calls.bam

3. **Basecall using the 'hac' model.**

**AI response:**

"To run basecalling using the 'hac' model, I will execute the following command:

This command uses the dorado tool with the hac model to perform basecalling on the input .pod5 file. The output will be a .bam file, which is then converted to a .fastq file using samtools."

**Terminal Request:**

dorado basecaller hac pod5/ > calls.bam && samtools fastq calls.bam > calls.fastq

4. **How can I enable duplex basecalling?**

**AI response:**

To enable duplex basecalling, you need to go to the **Basecalling settings** and check the box next to "Enable duplex" under the **Advanced options** section.

**Terminal Request:**

dorado duplex sup pod5/ > duplex_calls.bam && samtools fastq duplex_calls.bam > duplex_calls.fastq

5. **Convert 'basecalled.bam' to 'sequences.fastq'.**

**AI response:**

To convert the `basecalled.bam` file to a FASTQ file named `sequences.fastq`, you can use the following command:

This will extract the sequencing reads from the BAM file and output them in FASTQ format.

**Terminal Request:**

samtools fastq basecalled.bam > sequences.fastq

- **Quality Control & Filtering (NanoFilt & NanoPlot)**

6. **Filter my reads to keep only those with a Q-score greater than (> or =) 15.**

**AI response:**

To filter your reads and keep only those with a Q-score greater than or equal to 15, you can use the following command:

This will take the input file (input.fastq) and pass it through NanoFilt with the -q 10 option, which sets the minimum Q-score to 15. The output will be written to a new file called filtered.fastq.

Note: Make sure you have already run porechop on your input file to get the .fastq format.

Also, if you want to filter multiple files at once, you can use a loop:

for f in porechop_out/*.fastq; do cat "f"∣NanoFilt−q15>"f"∣NanoFilt−q15>"{f%.fastq}_filtered.fastq"; done

This will apply the same filtering to all .fastq files in the porechop_out/ directory.

**Terminal Request:**

cat input.fastq | NanoFilt -q 15 > filtered.fastq

7. **Keep reads with a length between 1450 and 1650 bp.**

**AI response:**

To keep reads with a length between 1450 and 1650 bp, you can use the following command:

This will filter out any reads that are shorter than 1450 bp or longer than 1650 bp.

Note: Make sure to replace input.fastq with your actual input file name.

**Terminal Request:**

NanoFilt -l 1450 --maxlength 1650 input.fastq > filtered.fastq

8. **Filter the FASTQ file for reads with quality above 15 and length above 1450.**

**AI response:**

To filter the FASTQ file, we will use `NanoFilt`. Here's the command:

This command will create a new file called filtered.fastq that contains only the reads with quality above 15 and length above 1450.

Note: Make sure to replace input.fastq with your actual input FASTQ file name.

**Terminal Request:**

cat input.fastq | NanoFilt -q 15 -l 1450 > filtered.fastq

9. **I need a QC report for the 'filtered_reads.fastq' file.**

**AI response:**

To generate a QC report for the `filtered_reads.fastq` file, I'll use NanoPlot to create a quality control report.

Here's the command:

This will produce a QC report in the qc_report directory with a hex plot and other metrics.

**Terminal Request:**

NanoPlot --fastq filtered_reads.fastq -o qc_report --legacy hex dot

10. **Run QC on all fastq files in the current directory.**

**AI response:**

To run quality control (QC) on all FastQ files in the current directory, I will use the following command:

This command tells NanoPlot to read all FastQ files in the current directory (*.fastq), generate a QC report named qc_report, and display it as a hex plot.

Please note that this will overwrite any existing file with the same name. If you want to save the output to a different location, please specify the path after -o.

Also, if you have multiple files with the same name but different extensions (e.g., file.fastq and file.fastq.gz), NanoPlot may not be able to read them correctly. In this case, you can use the following command instead:

NanoPlot --fastq .fastq -o qc_report --legacy hex dot

This will tell NanoPlot to read all files with the .fastq extension, regardless of their suffix.

Please let me know if you have any further questions or concerns!

**Terminal Request:**

NanoPlot --fastq *.fastq -o qc_report --legacy hex dot

- **Demultiplexing & Adapter Trimming (Porechop)**

11. **run BC10-BC20.**

**AI response:**

To run the basecalling for Barcode 10 to 20, I will use a loop to iterate through each barcode and execute the Dorado command.

This will create a new directory porechop_out and execute the Dorado command for each barcode from 10 to 20, saving the output in separate files.

Please note that this assumes you have already set up your input file path correctly. If not, please go to the Settings page and select the correct input file path.

Also, make sure you have enough disk space available, as the output files will be generated in the porechop_out directory.

Once the command is executed, you can proceed with demultiplexing using Porechop.

**Terminal Request:**

mkdir -p porechop_out && for i in {10..20}; do dorado basecaller sup pod5/ > calls_${i}.bam && samtools fastq calls_${i}.bam > calls_${i}.fastq; done

12. **Set the barcode threshold to 85 for demultiplexing.**

**AI response:**

To set the barcode threshold to 85, you can use the following command:

This will set the barcode threshold to 85 during demultiplexing.

Please note that this command assumes that your input file is named input.fastq and that you want to output the demultiplexed files in a directory named porechop_out. You can adjust these settings as needed for your specific use case.

**Terminal Request:**

porechop -i input.fastq -b porechop_out --barcode_threshold 85

13. **Run Porechop and save the output to 'demux_reads'.**

**AI response:**

To run Porechop, I will use the following command:

This will sort your sequencing reads into separate files based on their unique "Barcode" sequences. The output will be saved in a directory named 'demux_reads'.

Please note that you should replace input.fastq with the actual path to your input file.

Also, make sure that you have Porechop installed and available in your system's PATH. If not, you can download it from the official website or install it using a package manager like conda.

Once you run this command, Porechop will start processing your data and saving the output in the specified directory.

**Terminal Request:**

porechop -i input.fastq -b demux_reads/

14. **Demultiplex 'all_reads.fastq' into the 'barcodes' folder with a barcode_diff of 1.**

**AI response:**

To demultiplex your reads, I will use Porechop to separate them based on their unique barcode sequences.

Here's the command:
```bash
porechop -i all_reads.fastq -b barcodes/ --barcode_threshold 85 --barcode_diff 1
```
This will create a new folder called `barcodes` in your current working directory, and inside it, you'll find separate files for each barcode sequence.

Please note that the `--barcode_threshold` parameter is set to 85 by default. If you want to change this value, please let me know.

Also, keep in mind that Porechop will only demultiplex reads that have a perfect match with one of the barcodes in your library. If there are any reads that don't match any barcode, they will be left in the original file.

Let me know if you have any questions or need further assistance!

**Terminal Request:**

porechop -i all_reads.fastq -b barcodes/ --barcode_threshold 85 --barcode_diff 1

15. **Process 'input.fastq' with Porechop, outputting to a directory called 'pc_out'.**

**AI response:**

To process the file `input.fastq` with Porechop and output the results to a directory called `pc_out`, you can use the following command:

```bash
porechop -i input.fastq -b pc_out --barcode_threshold 85 --barcode_diff 1
```

This will demultiplex the reads in `input.fastq` based on their barcode sequences and output the results to a directory called `pc_out`. The `--barcode_threshold` option is set to 85, which means that only barcodes with at least 85% similarity to the reference barcode will be considered. The `--barcode_diff` option is set to 1, which means that reads with different barcodes will be output to separate files.

Please note that you need to have Porechop installed on your system and make sure it's in your PATH environment variable. If not, please refer to the installation instructions for Porechop.

**Terminal Request:**

porechop -i input.fastq -b pc_out --barcode_threshold 85 --barcode_diff 1

- **Taxonomy and Convert (emu & post_process.py)**

16. **I want to use the 18S rRNA gene database for taxonomy assignment.**

**AI response:**

To use the 18S rRNA gene database for taxonomy assignment, you'll need to select it from the Emu database dropdown menu.

1. Go to the **Taxonomy** tab in the settings.
2. Click on the **Emu Database** dropdown menu.
3. Select **18S_C_P_P** (or whichever 18S rRNA gene database is available).

This will ensure that Emu uses the correct database for taxonomy assignment based on your 18S rRNA gene sequences.

Please note that you'll also need to make sure that your input file contains 18S rRNA gene sequences. If not, you may want to consider using a different database or adjusting your analysis settings accordingly.

**Terminal Request:**

No generated

17. **Combine Taxonomy outputs in the 'taxonomy_out' directory to tax_id counts.**

**AI response:**

To combine the taxonomy outputs in the `taxonomy_out` directory to tax_id counts, you can use the following command:

```bash
emu combine-outputs taxonomy_out/ tax_id --counts
```

This will generate a file named `tax_id_counts.txt` in the current working directory, which contains the combined taxonomy results with tax_id as the identifier.

Please note that this assumes that the taxonomy outputs are in the correct format and that the `taxonomy_out` directory is correctly set up. If you encounter any issues or errors, please let me know!

**Terminal Request:**

emu combine-outputs taxonomy_out/ tax_id --counts

18. **Convert the file format for functional prediction.**

**AI response:**

To convert the file format for functional prediction, I will use the `post_process.py` script with the following flags:

This will generate a file in the correct format for PICRUSt2 functional prediction.

**Terminal Request:**

python3 post_process.py -i emu_results -d emu_db/16S_emu_default_database –picrust

19. **Generate a FAPROTAX compatible output from my taxonomy results.**

**AI response:**

To generate a FAPROTAX compatible output, I'll need to run the post-processing script with the `--faprotax` flag.

Here's the command:
```bash
python3 post_process.py -i emu_results -d emu_db/16S_emu_default_database --faprotax
```
This will generate a FAPROTAX compatible output in the `output_faprotax` directory. Please note that this may take some time to complete, depending on the size of your input data.

Also, please make sure that you have the necessary dependencies installed, including the `pandas` and `biopython` libraries.

If you'd like to run this command manually, you can do so by copying and pasting it into a terminal window. Alternatively, I can run it for you automatically if you'd prefer. Just let me know!

**Terminal Request:**

python3 post_process.py -i emu_results -d emu_db/16S_emu_default_database --faprotax

20. **Format 'taxonomy_out' for MicrobiomeAnalyst compatibility, selecting the 18S database.**

**AI response:**

To format `taxonomy_out` for MicrobiomeAnalyst compatibility using the 18S database, I will run the following command:

This command will convert the Emu output to a compatible format for MicrobiomeAnalyst, selecting the 18S database. Please wait while the process completes.

Note: Make sure you have the emu_db/18S_C_P_P database installed in your system and that it is correctly referenced in the post_process.py script.

Once the process finishes, you can find the formatted output in the same directory as the original taxonomy_out file.

**Terminal Request:**

python3 post_process.py -i emu_results/ -d /emu_db/18S_C_P_P --ma
